# Editorial: Insights in integrative bioinformatics–2021

**DOI:** 10.3389/fbinf.2023.1267370

**Published:** 2023-08-21

**Authors:** Zhi-Ping Liu

**Affiliations:** Department of Biomedical Engineering, School of Control Science and Engineering, Shandong University, Jinan, Shandong, China

**Keywords:** integrative bioinformatics, models and algorithms, omics data integration, data analytics algorithms and methods, editorial

The available high-throughput omics data, such as genomics, transcriptomics, proteomics and metabolomics, provide us unprecedented opportunity and challenge to decipher the corresponding global molecular profiles in cells. The need of data integration is urgent by developing integrative bioinformatics methods (Ebrahim et al., 2016). The bulk next-generation sequencing technique generated in the same or different phenotypes including experiments and conditions present multiple resources of amount of omics data. For instance, for a complex disease like breast cancer, multiple RNA-sequencing experiments for cancerous samples with paired adjacent normal tissues will request the integration of these transcriptomic datasets for obtaining consistent insights (Sammut et al., 2022).

This Research Topic aims to provide a Research Topic of integatve bioinformatics techniques for biomedical data integration. In “*MEMO: mass spectrometry-based sample vectorization to explore chemodiverse datasets*” Gaudry et al. proposed an approach called Memo for integrating mass spectrometry data. Memo captures the spectral diversity of complex samples to implement an efficient comparison of large amounts of samples without the need of a feature pre-alignment step. The efficiency of Memo was demonstrated on experiments about a large and chemodiverse sample clustering. Memo also demonstrates its superiority in computational time and other performance metrics.

In “*Application of network pharmacology in the study of mechanism of Chinese medicine in the treatment of ulcerative colitis: A review*” Zheng et al. proposed a summary of the applications of tranditional Chinese medicine (TCM) for the treatment of ulcerative colitis. They summarized the multiple TCM databases available for ulcerative colitis. The multiple datasets were organized via a network pharmacology framework. The TCM resources presented here will benefit for the monotherapy and compound therapy of ulcerative colitis.

In “*Algorithms to anonymize structured medical and healthcare data: A systematic review*” Sepas et al. proposed a systematic review of algorithms to anonymize structured medical and healthcare data (SMHD). The paper summarized and categorized different anonymization approaches for different types of SMHD, such as demographics, diagnosis codes and genomic data with sufficient levels of protection and utility. Further research is expected to build more efficient algorithms for the anonymization of SMHD in the biomedical big data era.

In “*Enhancer/gene relationships: Need for more reliable genome-wide reference sets*” Hoellinger et al. proposed a comparision study of the major methods available to detect the relationships between enhancer and gene. The identificaiton of enhancer-gene links will provide deep understanding of the cooperation between regulatory elements playing key roles in gene expression. In this work, the authors benchmarked three methods in the category of functional link methods. They concluded that it is urgent to propose new reliable and genome-wide reference data as well as the new bioinformatics methods for functional link identification between enhancer and gene.

These interesting papers shed lens for the integrative bioinformatics methods in diverse scenarios such as in genomics, proteomics, clinical and TCM data. These diverse applications indicates the integrative bioinformsitcs methods are important in multi-omics data analytics. These papers also demonstrate that we need proposed case-intensive and flexible data integration strategy and method for the available multi-omics data according to particular research purpose.
